# Supplementary figures and images for: Bayesian clustering with uncertain data
Source: PLoS Comput Biol. 2024 Sep 3;20(9):e1012301. doi: 10.1371/journal.pcbi.1012301 (PMC11398681; doi:10.1371/journal.pcbi.1012301)

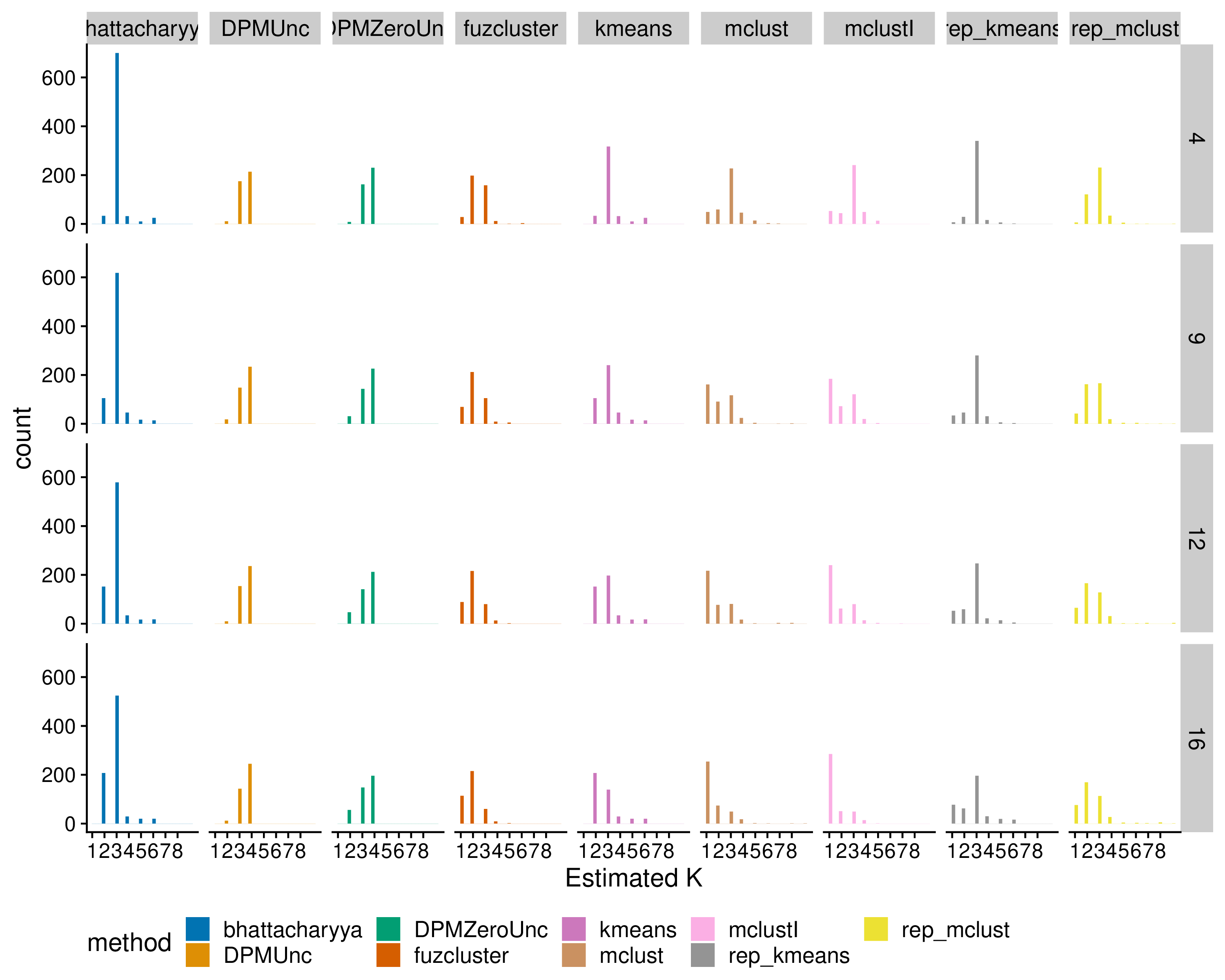

Supplement: S2 Fig — (TIFF) [file pcbi.1012301.s003.tiff]

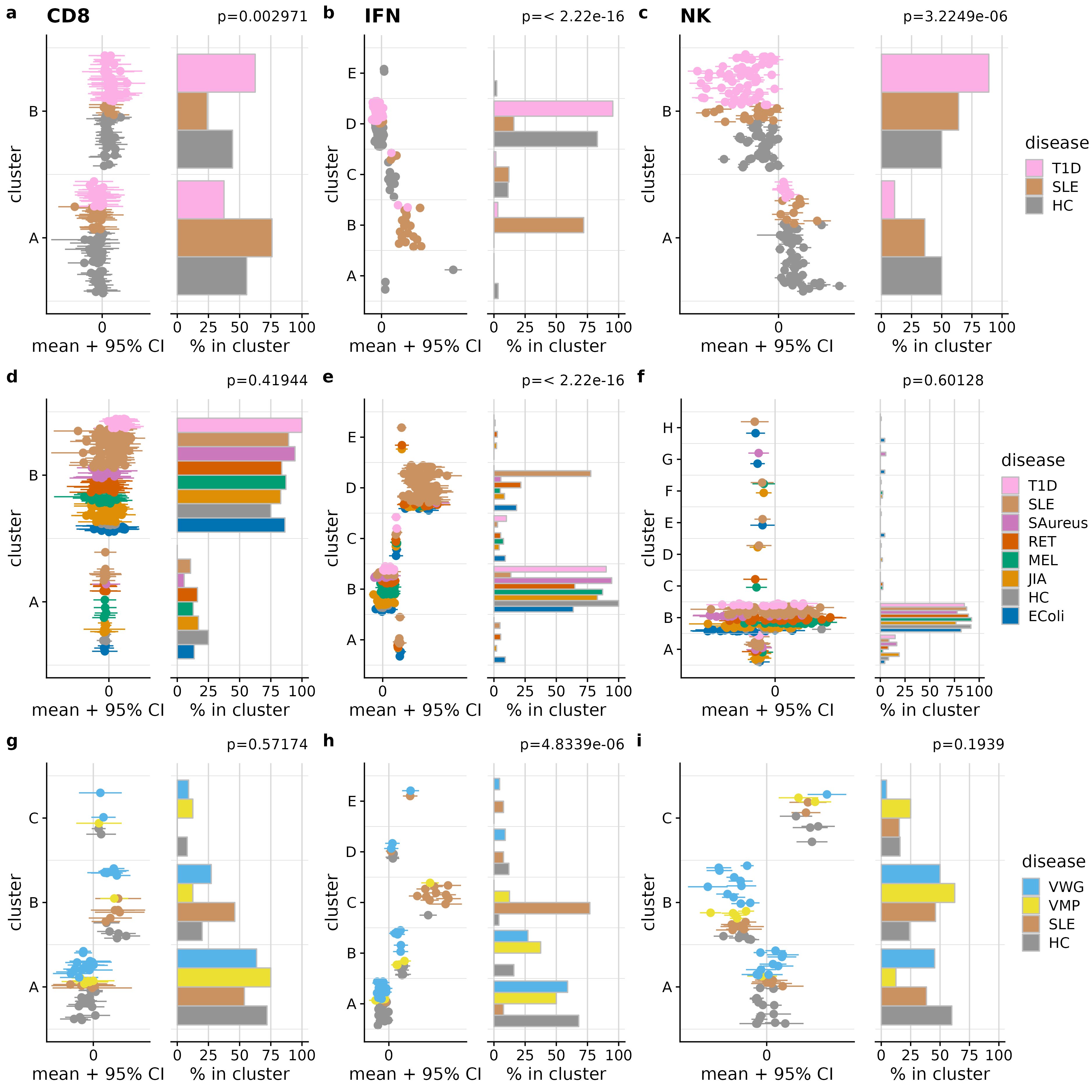

Supplement: S6 Fig — (a-c) Ferreira (d-f) Chaussabel (g-i) Lyons. In each panel, the left plot shows the observed data, and the right plot shows the fraction of individuals assigned to each cluster. The p value shown relates to the null hypothesis that cluster membership is independent of disease. This can be compared to Fig 4 where clustering used DPMUnc to allow for uncertainty. (TIFF) [file pcbi.1012301.s007.tiff]

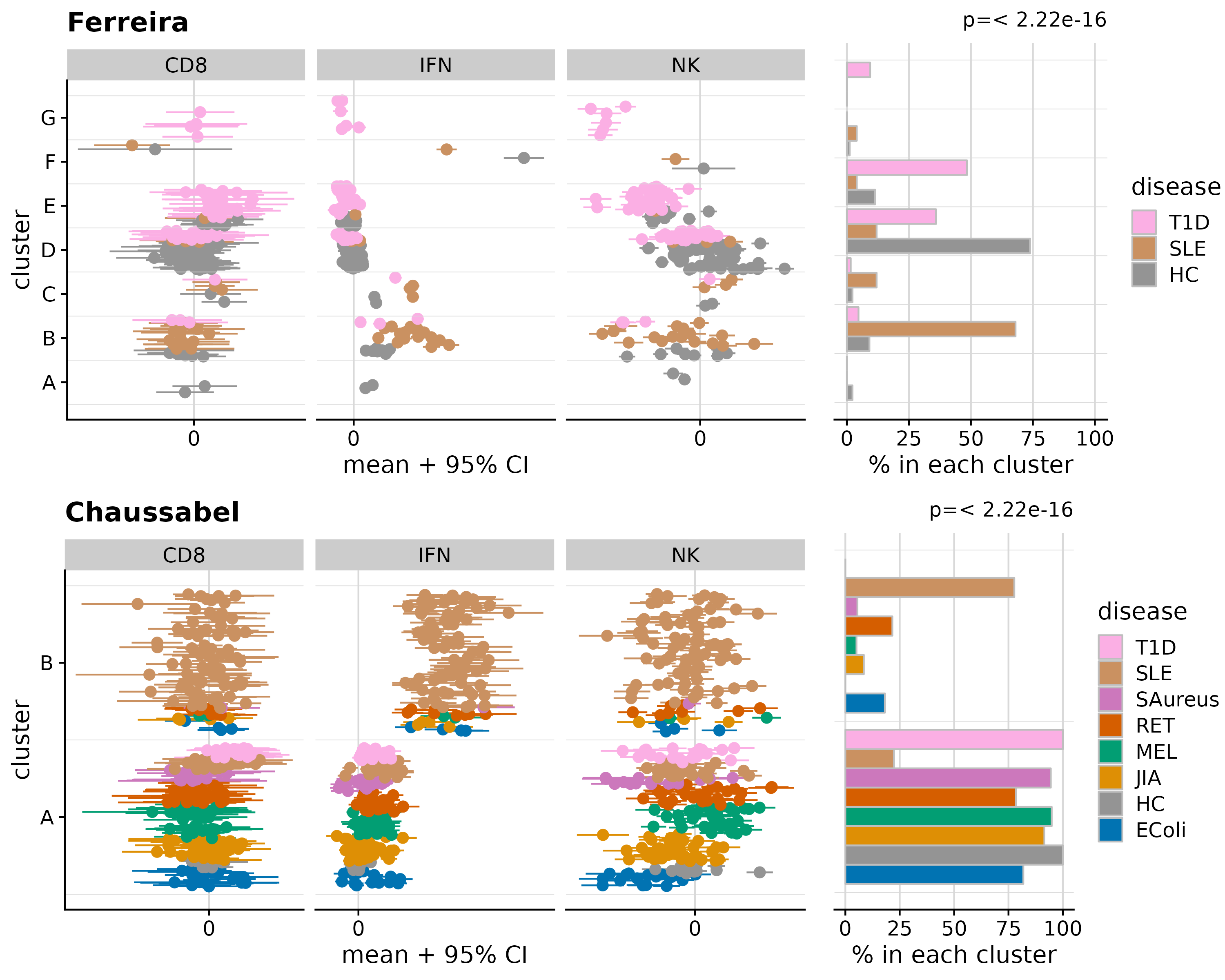

Supplement: S7 Fig — In each panel, the left plot shows the observed data, and the right plot shows the fraction of individuals assigned to each cluster. The p value shown relates to the null hypothesis that cluster membership is independent of disease. This can be compared to Fig 5 where clustering used DPMUnc to allow for uncertainty. (TIFF) [file pcbi.1012301.s008.tiff]

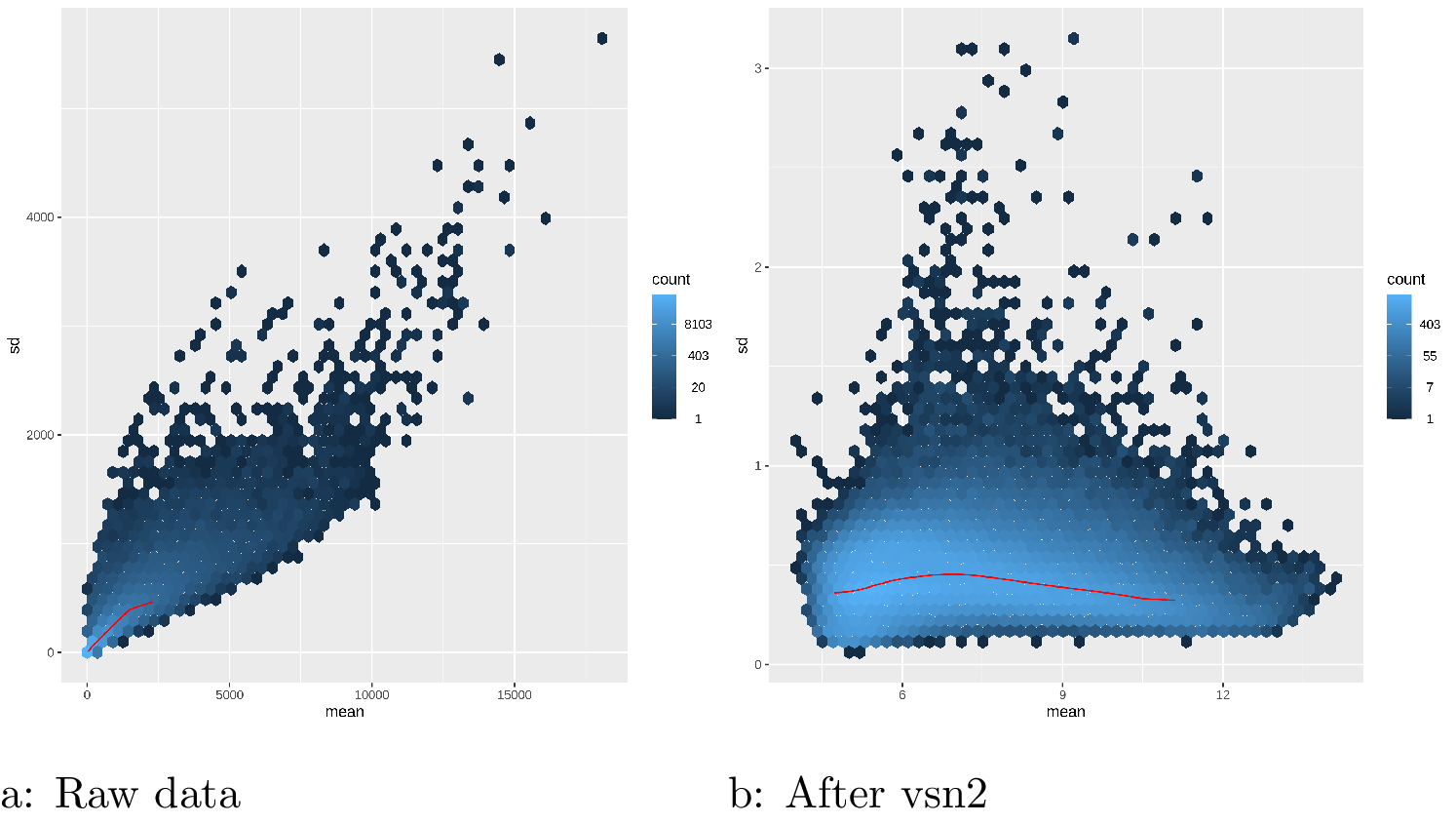

Supplement: S8 Fig — (TIFF) [file pcbi.1012301.s009.tiff]
