# Supplementary material for: Bayesian clustering with uncertain data
Source: PLoS Comput Biol. 2024 Sep 3;20(9):e1012301. doi: 10.1371/journal.pcbi.1012301 (PMC11398681; doi:10.1371/journal.pcbi.1012301)
Supplement: Attachment — Submitted filename: Comments to authors.pdf [file pcbi.1012301.s011.pdf]

Comments to the Authors

General

The authors proposed a method based on non-parametric Bayesian models to cluster samples, making use of the sample-wise uncertainty estimates. They evaluated their method in both simulated and real datasets and show that by utilizing sample-wise uncertainties, they can obtain results more consistent with ground truth or prior knowledge. For the gene expression dataset, they also proposed to group genes by gene signatures (gene sets) so as to reduce dimensions and to obtain uncertainty estimates.

The authors's approach of incorporating sample-wise uncertainties in modeling observations in clustering settings is an interesting idea and is well-motivated. However, except for this, the other designs or considerations for the probabilistic model are not presented in a clear and comprehensive manner. Also, the approach of grouping genes by signatures or gene sets is not novel. Lastly, the current benchmarking is limited and more evaluation results should be provided to support the claims.

Major comments

1. While it is clear that sample-wise uncertainties should be incorporated into clustering, it is not clear why the authors choose to use non-parametric Bayesian models. For example, it is not well elaborated the advantages of using Dirichlet Process model and why it fits the problem of interest.

2. The authors stated in the Results section that their part of the model generating the latent variables is equivalent to a Dirichlet Process Gaussian Mixture Model (DPGMM). So it seems the method is an extension of DGPMM, where they use DGPMM to generate latent variables and model the observations as samples from distributions parameterized by latent variables. The distributions for the observed data are also parameterized by uncertainty estimates. In this regard, the novelty of the method is mostly reflected in their way of parameterizing the sample distributions using sample-wise uncertainty estimates. However, the authors only compare with two baseline methods (k-means and Gaussian Mixture Models). These two methods are very different from the proposed model and is hard to dissect the exact cause to the differences in performances. To obtain more convincing results, the authors may consider include more relevant methods in comparison. For example, it is interesting to see how DGPMM without sample-wise uncertainty handling will perform.

3. In the Results section where the authors present the model, each observation x_i seems only represent a scalar value. However, the authors did run the method on multiple features

according to their results. It is not clear how this extends to multi-features/variables setting. For example, is each feature modeled independently using their own uncertainty estimate?

4. When clustering patients by gene expression, the authors proposed to group genes by gene signatures (gene sets) and use the summary statistics from a gene group as the feature and the estimate for the uncertainty. Again, it is a common practice to group genes by gene signatures and thus this approach is not novel.

5. Can the authors elaborate more on their choices of gene signatures? For example, why only three gene signatures? Are only three signatures available? How the quality of signatures affect the model's performance? In particular, here different signatures have different uncertainty estimates, which is different from the GWAS case where uncertainties are similar in different scores/features. In this case, how these differences in uncertainties affect the final results when running the proposed model using multiple features?

6. The authors ran the proposed method on multiple gene signatures and compared the results with those obtained from single signatures. However, it is not clear what leads to the differences in performance. Can the authors elaborate on this?

7. Throughout the evaluation stage, the proposed model was run on either one or three or thirteen features. These numbers may not well represent the number of features encountered in many bioinformatics problems. Can the authors elaborate on this? Is the proposed model has limitations and can only be run with a few features?

8. Related to the previous comment, the largest dataset used in the evaluation contains about three hundred samples. It is interesting to see how this method scales with number of samples and number of features. It would be better if runtime and memory analysis can be provided.
