# Supplementary material for: Bayesian clustering with uncertain data
Source: PLoS Comput Biol. 2024 Sep 3;20(9):e1012301. doi: 10.1371/journal.pcbi.1012301 (PMC11398681; doi:10.1371/journal.pcbi.1012301)
Supplement: Attachment — Submitted filename: review.pdf [file pcbi.1012301.s012.pdf]

Review of PCOMPBIOL-D-22-01876

"Bayesian Clustering with Uncertain Data"

Summary

The paper presents an algorithm based on Dirichlet process mixture model where it extends the standard model by including a measurement error on the level of indidivual samples. This is of great utility when the observations come with an observation-specific variance, such as when the observations come from another analysis with its associated noise or error. My overall view of the paper is positive, this is a useful extension to the standard clustering that uses measurement error when available. The authors also implemented the algorithm in an R package that is available for use. However, I believe the manuscript would benefit from a couple of changes:

1. The paper mentions "We introduce a novel procedure for generating a summary of a gene signature. . . " in the abstract. Does this correspond to the "Preprocessing gene expression data" section in the Methods part of the paper? This should be made more explicit in the text, perhaps including a pseudo-code to make the procedure for standardising signatures clearer. As a novel contribution, it should be more visible to the reader.
2. It would be useful if the paper highlighted the specific type of error it focuses on. The abstract is a little misleading, talking about data that have "associated uncertainty or measurement error". But uncertainty is included in any clustering algorithm as the cluster variance already! It should explicitly state that it looks at data that have **known** sample-specific uncertainty/measurement error.
3. When comparing the clustering on simulated data, the authors use `mclust` as one of the compared algorithms. The manuscript should specify what `mclust` settings were used, as the package offers multiple options for variance estimation (spherical, diagonal, elliptical, with varying volume and shape). In general, I found the comparison with mclust on the simulated datasets a little surprising. DPMCZeroUnc seems to correspond to Dirichlet process prior version of a standard Gaussian mixture model, i.e. the model behind `mclust`. The difference should only be in the number of clusters that are estimated by the algorithm - is that the case? Does `mclust` behave like DPMZeroUnc when the corrent number of clusters is selected (depending on the variance settings in `mclust`)?
4. What is the consistency of the DPUnc MCMC sampling? Do different runs result in the same estimated clusters or is it neccessary to run the algorithm several times to achieve a reasonable result?
5. The algorithm and analysis code is available openly on Github. There are a few minor issues that currently stop people from reproducinig the findings:

- The simulation scripts lack documentation. The scripts used for the GWAS analysis were presumably run on an HPC cluster, but lack environment specification. This makes it hard to replicate any of the experiments.
- The code used for analysis does not have a license (except for the R package), making it technically illegal for others to reuse.

Minor points

- Graphical model in Figure 1 should have (μ_k, ρ_k) instead of ϕ_k.
- Page 8, it would be better for the reader to explicitly say that the evaluation is being performed on 9 cases (for each signature for each dataset). Otherwise it is confusing where 7/9 comes from.
- Figure 4, the y axis in the plots looks like a numerical axis (1, 2, ..,). The numbers correspond to categorical variables, I would suggest changing the visualisation or renaming the clusters to A, B, C or similar.
