# Supplementary material for: Bayesian clustering with uncertain data
Source: PLoS Comput Biol. 2024 Sep 3;20(9):e1012301. doi: 10.1371/journal.pcbi.1012301 (PMC11398681; doi:10.1371/journal.pcbi.1012301)
Supplement: Attachment — Submitted filename: DPMunc-response1.pdf [file pcbi.1012301.s013.pdf]

Dear Editors,

Thank you very much for inviting us to revise our manuscript "Bayesian clustering with uncertain data" for consideration at PLOS Computational Biology. We have responded to all reviewers comments, and attach our revised manuscript and point by point response.

Reviewer 1

Authors extended the use of Dirichlet Process Gaussian Mixture models to incorporate the uncertainty associated with each sample. And they proposed Dirichlet Process Matrix Mixtures with Uncertainty (DPMUnc) as a nonparametric clustering framework. Authors conducted a simulation study that compared DPMUnc with existing methods, and further demonstrated that DPMUnc was effective in classifying immune-mediated diseases using GWAS summary statistics and clustering patients based on relevant gene signatures. The manuscript is well written and easy to follow, with detailed derivations included in the supplementary information. All the analysis pipelines are well-documented, ensuring the reproducibility of the study.

The use of uncertainty as a means of improving cluster assignment is an interesting problem, and the authors' proposed model appears to be theoretically sound. However, I have some concerns about the comparisons. Authors compared DPMUnc with Gaussian Mixture Model (GMM implementation) and k-means which are two baseline methods, while these two methods are not explicitly designed to handle data uncertainty, which may make the comparisons less persuasive. Moreover, certain details about the experiments need to be clarified.

Major concerns

1. The introduction section may benefit from a brief discussion on clustering the uncertain data, and authors could consider expanding the section to provide a more comprehensive overview. Additionally, there are alternative approaches clustering data with uncertainty that authors could include in the comparison. For instance, a few algorithms can be found via the following link: https://github.com/fgullo/jcludata.

Thank you for this suggestion, we were not aware of these algorithms. Unfortunately, we could not get them to work, reflecting partly some lack of documentation and mostly our lack of Java skills. We conducted a broader review of the literature, and identified three algorithms we could implement:

- fuzcluster (https://search.r-project.org/CRAN/refmans/sirt/html/fuzcluster.html)
- "representative clustering of uncertain data" (https://www.cs.uoi.gr/~nikos/kdd14.pdf), which we coded up in R
- hierarchical clustering using the Bhattacharyya distance

We have now broadened our introduction in the manuscript to include these algorithms, as well as those you identified and some others that we also failed to implement (again, written in Java):

"A variety of methods have been proposed to deal with uncertainty in observations when clustering, including calculating distance between observations accounting for uncertainty
10,
clustering representative objects sampled from the distribution associated with each uncertain
11,12 , and adapting the likelihood by integrating over the distribution associated observation with each uncertain observation 13."

The full list of algorithms we use for comparison is now given in the results:

"We then compared the performance of DPMUnc to a variety of clustering approaches which either do not account for uncertainty in observations: ˆ

- *kmeans 16 , one of the dominant non-parametric approaches that requires the number of clusters, K to be specified. We estimate K using the maximum gap statistic 17ˆ*
- *mclust 8 , based on a mixture of finite Gaussian models. K is chosen as the value which minimises the BIC of a range of models fitted*
- *DPMZeroUnc, which is DMPUnc with all sample uncertainties set to 0*

or do account for uncertainty

- *hierarchical clustering using the Bhattacharyya distance 18 . We choose K as TODO*
- *fuzcluster 12 which implements a similar model to DPMUnc in a maximum likelihood framework, albeit assuming the number of clusters K is known. We estimate K as for kmeans*
- *representative clustering of uncertain data 19 which provides a framework to extend a clustering method to uncertain data by sampling multiple examples of possible latent data, applying a clustering method to each and selecting a representative solution. We use representative clustering to extend kmeans and mclust for uncertain data"*

2. In the simulation study, I am wondering if mclust and kmeans were initialized from multiple starting points to avoid local optimal solutions.

Mclust internally initialises a single run from a hierarchical clustering model, and does not allow multiple initialisations. Kmeans used 25 initialisations. We have now made this clear in the Methods:

"We ran kmeans with 25 random starts to avoid convergence to a local optimum."

3. Authors clustered immune-mediated diseases with the GWAS statistics (Figure 3). DPMUnc and DPMZeroUnc disagree on the the diseases where the variance is relatively small, e.g., UKBB hyperthyroidism thyrotoxicosis, UKBB hypothyroidism myxoedema and UKBB pernicious anaemia. Could you elaborate on this observation?

The location of these three diseases is likely to be well estimated given the case numbers (and hence low variance). For other diseases in DPMUnc cluster A, the locations are estimated with more noise. DPMUnc estimates that with this noise, they could all belong in the same cluster together. On the other hand, DPMZeroUnc does not consider the noise, and sees the 3 "low noise" diseases as being similar enough to be placed in their own cluster. The centre of both clusters is similar. It is not that DMPZeroUnc sees these low noise diseases differently to DPMUnc, rather that it sees the higher noise diseases differently. Ie, DPMUnc allows that the noisier members of cluster A might be noisy samples from the same cluster as the low noise diseases, whereas DPMZeroUnc sees these noisy samples as too far from the tight low noise disease cluster and so places them in their own cluster with larger cluster variance.

4. Authors run DPMUnc to classify public gene expression dataset using relevant signatures. It would be helpful to include DPMZeroUnc and mclust to compare with DPMUnc and report corresponding ARI values. These could be included as part of the supplementary information. Another natural baseline here is to use mclust/kmeans to cluster all the genes included in the signature set without aggregation. This is a common practice to address similar clustering problems, and it would be compelling to show that DPMUnc can outperform this standard clustering pipeline.

We are not convinced that running multiple models on real data will produce useful inference. The different models will undoubtedly produce different results, and it would be beyond the scope of the current study to decide which is right. This was the point of the simulation study, where each method can be compared with a known ground truth. Our goal with the real data analyses was to demonstrate that DPMUnc can be applied to real data and produce interpretable output.

Minor concerns

1. Authors may consider broadening the scope of possible applications of the DPMUnc method as uncertainty commonly exists in most of the biological measurements. For example
(1) Authors could investigate if DPMUnc can improve upon the problem of clustering data based on multiple uncertain experts.
(2) Authors could experiment with network clustering as every edge/interaction was measured with a confidence score indicating uncertainty [1]
(3) Authors could explore the cell type annotation problem when dealing single-cell multiomic data. In this case, one cell could be assigned different cell types based on different modalities, e.g., RNA-seq and ATAC-seq. DPMUnc may be useful for integrating the annotation and clustering the single-cell data more accurately [2].

Thank you for these suggestions, and these are interesting examples. It is important to note that DPMUnc models data within a cluster as being distributed according to a multivariate Gaussian distribution, which may not be appropriate for all of the suggested applications, but we have now included them in our extended introduction (see below).

"Examples include data generated by multiple uncertain experts [9] , network clustering where edges have an associated score indicating uncertainty, or the cell type annotation problem in single-cell multiomic data, where different omics layers may assign cells to different clusters. In this work, we focus on quantitative data with associated uncertainty, which may arise for example with random measurement error or the use of summary measures such as the average of three blood pressure readings."

2. The uncertainty variable (variance) only influences the latent variable sampling step. Therefore, DPMZeroUnc should be equivalent as the standard Dirichlet Process Gaussian Mixture. It may be beneficial for authors to compare DPMZeroUnc with existing implementations of Dirichlet Process Gaussian Mixture models, such as sklearn, to verify if the convergence behavior is similar.

sklearn.mixture.BayesianGaussianMixture is not the same model as ours:

- independent normal priors on mean and precision of the cluster locations vs normal-gamma prior for DPMUnc
- fixed value for alpha in DP vs alpha ~ Gamma(3,4) in DPMUnc
- Inference performed by variational methods rather than MCMC

and, as might therefore be expected, sklearn does not produce the same solution as DPMUnc, and therefore we cannot compare convergence behaviours.

3. Sampling algorithms in general are computationally intensive. Authors can include some basic statistics on the running time and include a short discussion in the last section.

We have added a section on timings to the supplementary which shows that DPMUnc runs in.

4. Supplementary figures S1 and S2 are really intuitive and helpful. It would enhance the clarity of the figures and make the respective legends more reader friendly if different panels can be labeled by letters.

We have now done this.

Reference
1. Jonsson, Pall F., and Paul A. Bates. "Global topological features of cancer proteins in the human interactome." Bioinformatics 22.18 (2006): 2291-2297.
2. Azimuth, https://azimuth.hubmapconsortium.org/

Reviewer 2

> Comments to the Authors General The authors proposed a method based on non-parametric Bayesian models to cluster samples, making use of the sample-wise uncertainty estimates. They evaluated their method in both simulated and real datasets and show that by utilizing sample-wise uncertainties, they can obtain results more consistent with ground truth or prior knowledge. For the gene expression dataset, they also proposed to group genes by gene signatures (gene sets) so as to reduce dimensions and to obtain uncertainty estimates. The authors's approach of incorporating sample-wise uncertainties in modeling observations in clustering settings is an interesting idea and is well-motivated. However, except for this, the other designs or considerations for the probabilistic model are not presented in a clear and comprehensive manner. Also, the approach of grouping genes by signatures or gene sets is not novel. Lastly, the current benchmarking is limited and more evaluation results should be provided to support the claims.
>
> Major comments
>
> 1. While it is clear that sample-wise uncertainties should be incorporated into clustering, it is not clear why the authors choose to use non-parametric Bayesian models. For example, it is not well elaborated the advantages of using Dirichlet Process model and why it fits the problem of interest.

We have now described our rationale for choosing a DP model for this problem in the introduction:

"Within clustering approaches generally, the Bayesian methods offer some advantages, in their ability to convey uncertainty about the overall clustering, and particularly to simultaneously infer the number of clusters, K."

> 2. The authors stated in the Results section that their part of the model generating the latent variables is equivalent to a Dirichlet Process Gaussian Mixture Model (DPGMM). So it seems the method is an extension of DGPMM, where they use DGPMM to generate latent variables and model the observations as samples from distributions parameterized by latent variables. The distributions for the observed data are also parameterized by uncertainty estimates. In this regard, the novelty of the method is mostly reflected in their way of parameterizing the sample distributions using sample-wise uncertainty estimates. However, the authors only compare with two baseline methods (k-means and Gaussian Mixture Models). These two methods are very different from the proposed model and is hard to dissect the exact cause to the differences in performances. To obtain more convincing results, the authors may consider include more relevant methods in comparison. For example, it is interesting to see how DGPMM without sample-wise uncertainty handling will perform.

DGPMM without sample-wise uncertainty is covered by DPMZeroUnc, where we use our DPGMM but set all the sample-wise uncertainty values to zero. Importantly, this means any differences in performance between DPMUnc and DPMZeroUnc are solely due to the

in/exclusion of the uncertainty information and not any differences in sampling algorithms. We have also expanded our baseline methods considerably:

"We then compared the performance of DPMUnc to a variety of clustering approaches which either do not account for uncertainty in observations: ̂

- *kmeans 16 , one of the dominant non-parametric approaches that requires the number of clusters, K to be specified. We estimate K using We estimate K using the maximum gap statistic 17̂*
- *mclust 8 , based on a mixture of finite Gaussian models. K is chosen as the value which minimises the BIC of a range of models fitted*
- *DPMZeroUnc, which is DMPUnc with all sample uncertainties set to 0*

or do account for uncertainty

- *hierarchical clustering using the Bhattacharyya distance 18 . We choose K as TODO*
- *fuzcluster 12 which implements a similar model to DPMUnc in a maximum likelihood framework, albeit assuming the number of clusters K is known. We estimate K as for kmeans*
- *representative clustering of uncertain data 19 which provides a framework to extend a clustering method to uncertain data by sampling multiple examples of possible latent data, applying a clustering method to each and selecting a representative solution. We use representative clustering to extend kmeans and mclust for uncertain data"*

The greatest determinant of performance appears to be the ability to correctly infer K:

"The methods differed most in their ability to infer K. When the true K = 3, DPMUnc and DPMZeroUnc tended to select K = 3 or K = 4, whilst kmeans and, to a greater extent, mclust, tended to merge clusters and underestimate K, often selecting K = 1 as cluster noise increased (Supplementary Figure S7 FIXME What is up with number of examples for bhatt?). Supplying the true value of K to methods where possible (TODO list them once image fixed) improved accuracy, often to greater than DPMUnc (which infers K)."

> 3. In the Results section where the authors present the model, each observation xi seems only represent a scalar value. However, the authors did run the method on multiple features 1 according to their results. It is not clear how this extends to multi-features/variables setting. For example, is each feature modeled independently using their own uncertainty estimate?

Yes, exactly. We have assumed independence between features and do not attempt to model a variance-covariance matrix, equivalent to assuming a diagonal variance-covariance matrix in mclust. We now make that clear at the end of the first Results section which describes the model:

"This model is stated for univariate data but can easily be extended for multivariate data, using the assumption that dimensions are independent and have the same distribution, so that the same hyperparameters can be used for every dimension. For multivariate data with p dimensions, the variables x_i, z_i, μ_k and ρ_k^2 would all be p-dimensional vectors and the corresponding distributions set out in the Supplementary Information may be used to sample

each dimension independently."

4. When clustering patients by gene expression, the authors proposed to group genes by gene signatures (gene sets) and use the summary statistics from a gene group as the feature and the estimate for the uncertainty. Again, it is a common practice to group genes by gene signatures and thus this approach is not novel.

We agree that it is common to group genes by gene signatures, the novelty here comes from including an estimate of the variability of expression across genes in the set. We have now made this clear in the Abstract, which is the only place we claim novelty:

" We first introduce a novel procedure for generating a summary of a gene signature on a dataset different to the one where it was discovered, which incorporates a measure of the variability in expression across signature genes within each individual."

5. Can the authors elaborate more on their choices of gene signatures? For example, why only three gene signatures? Are only three signatures available? How the quality of signatures affect the model's performance? In particular, here different signatures have different uncertainty estimates, which is different from the GWAS case where uncertainties are similar in different scores/features. In this case, how these differences in uncertainties affect the final results when running the proposed model using multiple features?

We chose these gene signatures as representative examples familiar to us from working within the field of immune-mediated diseases. We did perform a small literature search to expand the number of gene signatures, but did not manage to find additional signatures that we thought would be relevant to the example gene expression datasets we had available for their application. Throughout, we consider settings in which uncertainty estimates are provided with each data point, i.e. where the uncertainty estimates form part of the observed data. Considering both the GWAS and gene signature cases allows us to demonstrate the utility of DPMUnc in settings where the distributions of the uncertainty estimates may be different, which is also assessed through different simulation scenarios in our simulation study.

6. The authors ran the proposed method on multiple gene signatures and compared the results with those obtained from single signatures. However, it is not clear what leads to the differences in performance. Can the authors elaborate on this?

We believe that the different signatures might relate to different biological processes involved in development or progression of IMD. Thus, clustering by different individual signatures might be expected to lead to different clusterings of samples. The joint clustering is one way to resolve these differences, producing a single clustering which captures input from potentially multiple biological processes.

7. Throughout the evaluation stage, the proposed model was run on either one or three or thirteen features. These numbers may not well represent the number of

features encountered in many bioinformatics problems. Can the authors elaborate on this? Is the proposed model has limitations and can only be run with a few features?

We expect that 13 is close to an upper limit for the application of DPMunc, partly because of the assumption that features are independent. In the GWAS case, we can assume independence because the features are derived through PCA, and in the gene expression case because there is no gene overlap between signatures. We have now commented on this in the discussion:

"We note that because DPMUnc assumes independence between features, its use is likely to be restricted to data that is relatively low dimensional. In the real data examples shown here, we assume there is limited dependence between the genetic features as these were derived using PCA and between the transcriptomic features because there is no overlap of genes between the different signatures."

8. Related to the previous comment, the largest dataset used in the evaluation contains about three hundred samples. It is interesting to see how this method scales with number of samples and number of features. It would be better if runtime and memory analysis can be provided.

We now provide a summary of the timing with increasing number of samples and dimensions in the Supplementary Information.

"We considered how DPMUnc runtimes scaled with the number of samples and the number of features. For low dimensional data, runtimes appeared to increase linearly with sample size, although there was some variability in this for higher dimensional data. Timing appears to increase exponentially with number of dimensions, although all runs (4 parallel chains, run for XX on a single CPU) completed in around 100 seconds even for 200 samples and 18 dimensions."

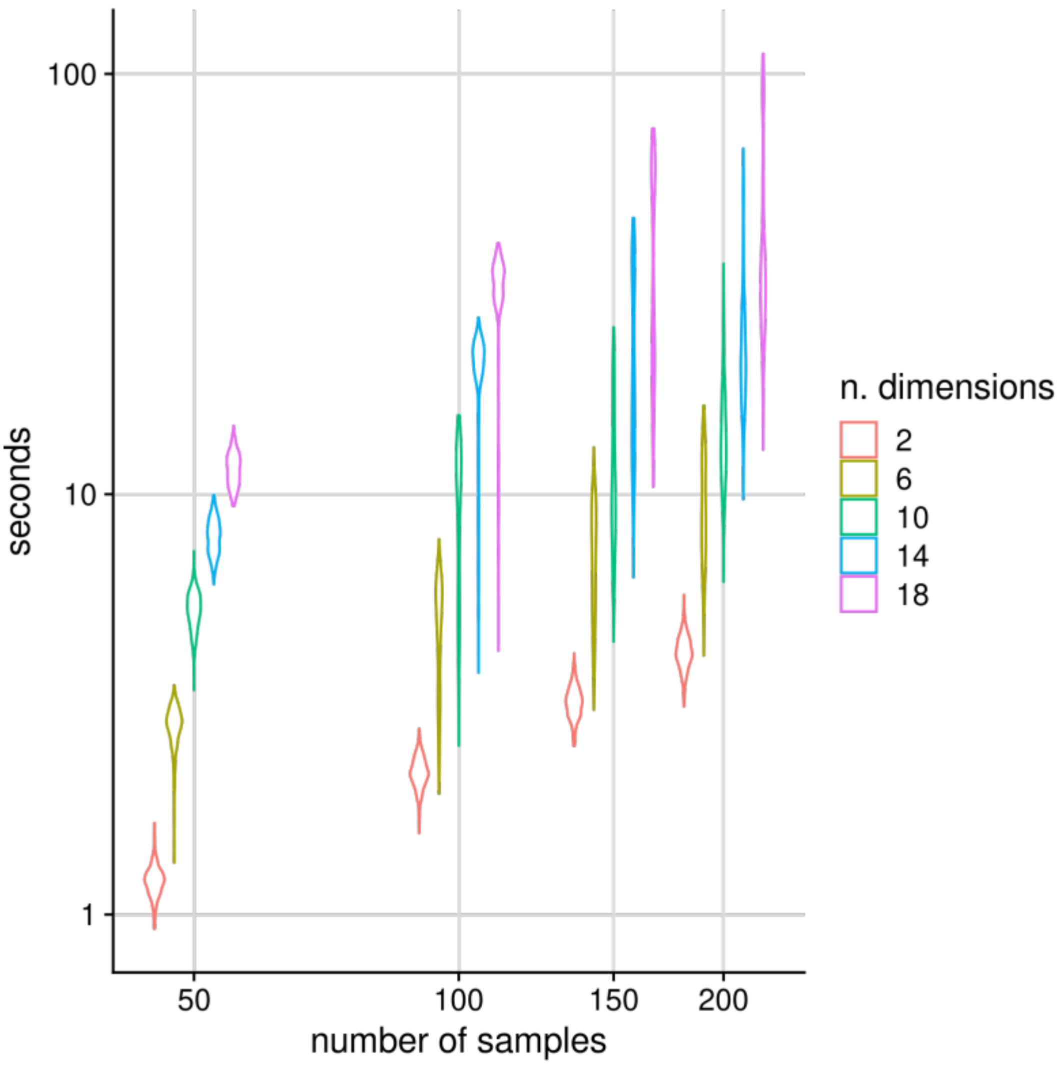

Supplementary Figure S3: Distribution of runtimes for DPMUnc as the number of samples and features increases

Reviewer 3

> **Summary** The paper presents an algorithm based on Dirichlet process mixture model where it extends the standard model by including a measurement error on the level of indidivual samples. This is of great utility when the observations come with an observation-specific variance, such as when the observations come from another analysis with its associated noise or error. My overall view of the paper is positive, this is a useful extension to the standard clustering that uses measurement error when available. The authors also implemented the algorithm in an R package that is available for use. However, I believe the manuscript would benefit from a couple of changes:
>
> 1. The paper mentions “We introduce a novel procedure for generating a summary of a gene signature. . . ” in the abstract. Does this correspond to the “Preprocessing gene expression data” section in the Methods part of the paper? This should be made more explicit in the text, perhaps including a pseudo-code to make the procedure for standardising signatures clearer. As a novel contribution, it should be more visible to the reader.

As we say in response to reviewer 2, the novelty is about using two measures to summarise the expression across genes rather than one. We now make this clearer in Methods

“The selected signature genes are listed in full in Table S1 and were summarised for each sample by two values, the mean and the variance of the normalised expression value, with the summary measures taken across all genes in a signature.”

> 2. It would be useful if the paper highlighted the specific type of error it focuses on. The abstract is a little misleading, talking about data that have “associated uncertainty or measurement error”. But uncertainty is included in any clustering algorithm as the cluster variance already! It should explicitly state that it looks at data that have known sample-specific uncertainty/measurement error.

Thank you, we agree this clarification is useful. We have added the following text to the second paragraph of the introduction:

“ To be clear, clustering methods already capture one source of uncertainty as the cluster variance, which describes variability in the location of different objects in the same cluster. Here, when we discuss uncertainty, we will focus on uncertainty attached to each observation, which may vary between observations and/or features. In this case, cluster variance would describe the variability of location of the latent observations which can be thought to represent the ``true'' locations of the objects whose location we have observed with some measured uncertainty.”

> 3. When comparing the clustering on simulated data, the authors use mclust as one of the compared algorithms. The manuscript should specify what mclust settings were used, as the package offers multiple options for variance estimation (spherical, diagonal, elliptical, with varying volume and shape). In general, I found the

comparison with mclust on the simulated datasets a little surprising. DPMCZeroUnc seems to correspond to Dirichlet process prior version of a standard Gaussian mixture model, i.e. the model behind mclust. The difference should only be in the number of clusters that are estimated by the algorithm - is that the case? Does mclust behave like DPMZeroUnc when the corrent number of clusters is selected (depending on the variance settings in mclust)?

We have clarified that

"We ran mclust with default settings and chose K by optimising the Bayesian Information Criterion as recommended by the authors."

We believe lesser performance of mclust relates to its tendency to underestimate K, particularly as uncertainty increases when it often chose K=1. We found that fixing K to its true value led to substantial improvements in performance of mclust, often exceeding that of DPMUnc even though mclust was not accounting for uncertainty. Thus we believe that the behaviour of the DP in allowing DPMUnc to estimate K is an important part of its performance.

4. What is the consistency of the DPUnc MCMC sampling? Do different runs result in the same estimated clusters or is it neccessary to run the algorithm several times to achieve a reasonable result?

We have used 4 parallel chains for every analysis, and combined the draws (after excluding burn off) for all simulated results shown. While we recognise that in real data analysis we would always check the convergence diagnostics, this is not practical for simulated data because of the number of runs. We therefore TODO

5. The algorithm and analysis code is available openly on Github. There are a few minor issues that currently stop people from reproducinig the findings:
• The simulation scripts lack documentation. The scripts used for the GWAS analysis were presumably run on an HPC cluster, but lack environment specification. This makes it hard to replicate any of the experiments.

We have added a Readme.md file to the repository which includes description of the HPC, and the versions of R and snakemake used to run the scripts. We are happy to add any further information if more is needed, but we are not clear what a complete environment specification might be.

• The code used for analysis does not have a license (except for the R package), making it technically illegal for others to reuse.

We have now added GPL3 license where none existed.

Minor points

• Graphical model in Figure 1 should have (μk, ρk) instead of ϕk.

Done

- Page 8, it would be better for the reader to explicitly say that the evaluation is being performed on 9 cases (for each signature for each dataset). Otherwise it is confusing where 7/9 comes from.

Done

"We first used DPMUnc to cluster three datasets by each of the three individual signatures, i.e. nine clustering tasks overall, and found clusters in each dataset which showed association with disease in the majority of cases (7/9)."

- Figure 4, the y axis in the plots looks like a numerical axis (1, 2, ..,). The numbers correspond to categorical variables, I would suggest changing the visualisation or renaming the clusters to A, B, C or similar. 2

Done
