# Supplementary material for: Bayesian clustering with uncertain data
Source: PLoS Comput Biol. 2024 Sep 3;20(9):e1012301. doi: 10.1371/journal.pcbi.1012301 (PMC11398681; doi:10.1371/journal.pcbi.1012301)
Supplement: Attachment — Submitted filename: Comments to the Authors.pdf [file pcbi.1012301.s014.pdf]

Comments to the Authors

General

The manuscript has now been improved. I have one remaining concern regarding the methods evaluation part. Also, some part of the figures are missing (see Minor comments).

Major comments

1. The authors claimed that they proposed a novel way to combine genes into gene sets by also considering the variability in expression across genes and use this as the uncertainty estimate in their modeling. It is interesting to see how this novel approach benefits the clustering. For example, will their proposed approach perform better, comparing to approaches which do not account for variability in expression? For now, the authors do not include any comparison in section "Clustering patients by gene expression signatures" where they implemented their approach on group genes in gene sets. It would be more convincing if more comparisons can be included for this task.

Minor comments

1. It seems Fig. 1 is missing.
2. Several parts of Fig. 2 are missing (e.g. the legend).
