# Supplementary material for: Bayesian clustering with uncertain data
Source: PLoS Comput Biol. 2024 Sep 3;20(9):e1012301. doi: 10.1371/journal.pcbi.1012301 (PMC11398681; doi:10.1371/journal.pcbi.1012301)
Supplement: Attachment — Submitted filename: DPMUnc_response2.pdf [file pcbi.1012301.s015.pdf]

Reviewer #1:

I appreciate the authors' comprehensive response, which effectively addressed most of my concerns. Overall, my impression is positive. While I acknowledge the arguments presented in response to my primary concern #4, I would like to underscore the importance of comparing the diverse outcomes yielded by different methods on the real datasets. Simulation experiments offer a controlled and constrained environment for showcasing the superior performance of the proposed method. The objective of real experiments, however, is not to identify the "best" model, given the potential variability in their outputs. Rather, it is to address the question: what novel insights can be exclusively derived through the application of DPMUnc as opposed to alternative approaches? This inquiry aims to provide an intuitive grasp of DPMUnc's behavior within a practical context. I hope this clarification will alleviate any confusion stemming from my initial inquiry.

We have now run DPMUnc without uncertainty to compare the results to standard DPMUnc (Fig SX and Fig SX). We described the results for single dataset clustering on page 10:

“Clustering using DPMUnc without uncertainty (DPMZeroUnc) produced very similar clusterings for 7/9 examples, but very different in two cases (S6 Fig). For SigCD8 in the Ferreira dataset, clustering without uncertainty produced two clusters instead of six, while for SigNK in Ferreira, it found eight instead of two. Thus we saw no systematic difference in number of clusters by the two methods. However, while both examples produced clusterings that associated significantly with disease when including uncertainty ($p < 10^{-8}$), neither was associated with disease when uncertainty was ignored, suggesting that for these two examples accounting for uncertainty was required to reveal biologically meaningful clusterings.“

We discussed multiple dataset clusterings on page 11:

“When uncertainty was ignored (DPMZeroUnc), the Fereirra results were broadly similar (S7 Fig). Note, however, that we would not have considered multiple signature clustering on the basis of the DPMZeroUnc single dataset clusterings, as only one signature was significantly associated with disease. However, the Chaussabel dataset was clustered into only two groups, defined by IFN alone, with no distinction between samples with lower Sig NK values (groups F–I in Figure 5).“

We also referenced this analysis in the Discussion:

“Our proposed method of summarising a gene expression across a signature extends the possible uses of signatures, and since the signature may be weaker on a dataset with more complex structure, it is crucial to take the variability of the signature into account, which DPMUnc allows. In particular, it allows us to cluster patients

simultaneously according to multiple signatures."

Upon careful review of the response, I observed several placeholders labeled as TODO within the response document. I strongly recommend a thorough examination prior to finalizing and submitting the document.

Thank you, we have very carefully reviewed the final submission and believe it is now free of any placeholders.

Reviewer #2:

General

The manuscript has now been improved. I have one remaining concern regarding the methods evaluation part. Also, some part of the figures are missing (see Minor comments).

Major comments

1. The authors claimed that they proposed a novel way to combine genes into gene sets by also considering the variability in expression across genes and use this as the uncertainty estimate in their modeling. It is interesting to see how this novel approach benefits the clustering. For example, will their proposed approach perform better, comparing to approaches which do not account for variability in expression? For now, the authors do not include any comparison in section "Clustering patients by gene expression signatures" where they implemented their approach on group genes in gene sets. It would be more convincing if more comparisons can be included for this task.

We have added a comparison to DPZeroMunc - see response to reviewer 1 above. This seemed the most relevant comparison since the model is the same, so we are explicitly examining the effect of including uncertainty or not, rather than allowing that comparison to be confounded by different clustering models.

Minor comments

1. It seems Fig. 1 is missing.

Fig1 was included in the pdf, but not as a standalone file. Now corrected.

2. Several parts of Fig. 2 are missing (e.g. the legend).

Now corrected.
